# Epigenetic mechanisms of retroviral regulation: a comparative review

**DOI:** 10.1186/s13072-025-00659-6

**Published:** 2026-02-03

**Authors:** So Youn Shin, Momtahina Tahmida, Nadejda Beliakova-Bethell, Sarah A. LaMere

**Affiliations:** 1 Catholic Kwandong University School of Medicine, Gangneung-si, Gangwon-do South Korea; 2 Department of Medicine, University of California San Diego, 9500 Gilman Dr., La Jolla, CA 92093 USA; 3 VA San Diego Healthcare System, San Diego, CA USA

**Keywords:** Retrovirus, Epigenetics, Gammaretrovirus, HIV, Murine leukemia virus, Latency

## Abstract

Major pharmacologic advances the past few decades have transformed infection with Human Immunodeficiency Virus (HIV) from a fatal disease into a chronic, manageable condition for people with access to antiretroviral therapy (ART). However, a cure remains elusive because HIV persists in a latent state throughout the body, evading immune clearance after ART suppression. Our understanding of HIV latency has made tremendous strides the past few decades, but the specific epigenetic mechanisms underlying latency are still being elucidated. Insights might be gained from simpler retroviruses capable of endogenization, such as the gammaretrovirus Murine Leukemia Virus (MLV). Most vertebrates, including humans, exhibit evidence for ancient retroviral infections that have been epigenetically silenced during early embryogenesis, offering natural modes of viral repression. This review summarizes our current understanding of epigenetic and epitranscriptomic silencing of HIV-1, highlighting parallels and contrasts with MLV and other retroviruses throughout the animal kingdom. We also discuss epigenetic mechanisms of pre-integration latency and T cell-mediated control, made possible through comparative studies of retroviral infections in other species. Finally, we propose how insights from other retroviruses might inform strategies for durable HIV-1 suppression.

## Background

Epigenetic mechanisms are the cellular processes that regulate gene expression and determine how genes are turned "on" or "off" without altering the underlying DNA sequence. Understanding epigenetic mechanisms controlling HIV infection is crucial to comprehending retroviral persistence, shedding light on viral eradication, and developing rational therapeutic interventions. Although the literature still only scratches the surface of the topic, comprehensive and extensive research on epigenetic mechanisms in HIV-1 infection has been published, especially in post-integration latency. There are several conserved key host epigenetic mechanisms known to impact retroviral latency, including DNA methylation, histone modifications, and non-coding RNA molecules. However, epigenetic mechanisms of pre-integration latency or T cell control of the virus are less studied in HIV-1 infection. Epigenetic control of non-human retroviruses is even less explored, despite the fact they can provide a window into pertinent mechanisms for understanding HIV-1. For example, studies published in MLV, a member of the gammaretrovirus family, suggest a possible epigenetic mechanism of silencing the pre-integrated viral DNA [1–3].

Gammaretroviruses are known as 'simple' retroviruses due to the lack of accessory proteins characteristic of the lentiviruses such as HIV. Because of their propensity to endogenize by infecting germ cells, silencing mechanisms of naturally occurring gammaretroviruses such as MLV have been extensively studied in embryonic stem cells (ESCs), as transcriptional machinery is shut down shortly as a host molecular defense against these genome invaders, calling into question whether some of these mechanisms might be useful in suppressing HIV. Indeed, several of them overlap with known mechanisms of HIV-1 latency, highlighting the need to harness those that have been characterized for MLV but entirely unexplored for HIV. Differences in tropism, replication, and integration preference between these retroviral classes might provide some insight into their transcriptional control (Table 1).

**Table 1:** Comparison of basic characteristics of MLV and HIV-1

Characteristic	MLV	HIV-1
Encoded proteins	Gag/Pol/Env	Gag/Pol/Env + 2 regulatory and 4 accessory proteins
PIC	Does not enter the intact nucleus in rapidly dividing cells	Enters through nuclear pores
Susceptible cells	Infects primarily dividing cells	Infects resting and dividing cells
Reverse transcription location	In cytoplasm	Begins in the cytoplasm but completes near or within the nucleus
Integration site	promoter-proximal	transcriptional units, promotor-distant
Cytoplasmic unspliced RNA pool	Two distinct populations, one for translation, and one for viral genome packaging	One large pool from which translation and genome packaging occurs

Ref. [4–15]

Gammaretroviruses, who have endogenous counterparts such as Feline Leukemia Virus (FeLV) and the Koala Retrovirus (KoRV), have been characterized in terms of their control by Krüppel-associated box-containing (KRAB) zinc finger proteins and non-coding RNAs. Furthermore, studies done in natural lentiviral infections, such as Simian Immunodeficiency Virus (SIV) infection in African Green Monkeys and Feline immunodeficiency Virus (FIV) in domestic cats, suggest epigenetic mechanisms of T cell regulation impacting retroviral infection. Several recent reviews have examined epigenetic regulation of HIV-1 [16–18] and that of the simpler retroviruses [19, 20]. However, to our knowledge no comprehensive comparative review currently exists that examines similarities and differences in this regulation between the different types of retroviruses. This review focuses primarily on epigenetic and epitranscriptomic mechanisms regulating retroviral latency, while briefly summarizing T cell–mediated influences as complementary modes of proviral control. We first provide an overview of our current understanding of the epigenetic mechanisms involved in the silencing of HIV-1. We next compare and contrast recent studies regarding epigenetic mechanisms of viral control in some of the non-human retroviruses, particularly those that are capable of endogenizing. These comparative insights highlight three complementary layers of retroviral regulation that remain incompletely understood in HIV-1 but are illuminated by studies in other systems—including the koala retrovirus (KoRV), a uniquely informative model of an endogenizing gammaretrovirus: (1) pre-integration latency, (2) post-integration latency, and (3) T cell–mediated control of proviral expression. By examining these across retroviral classes, we aim to identify conserved principles and mechanistic distinctions that may inform strategies for durable HIV-1 silencing.

## Methods

Literature searches were conducted in PubMed using keywords (e.g. HIV, MLV, retroviral epigenetics, histone modifications, DNA methylation, etc.). Articles addressing epigenetic modifications relevant to proviral control both pre- and post-integration were selected for review. Figures were created in Biorender.com.

## Epigenetic mechanisms of HIV-1 latency: a framework for comparison

Our initial discussion begins with HIV-1 latency, as its mechanisms of epigenetic control are the most extensively characterized, providing a framework for comparison with other retroviral systems discussed in later sections. Latency refers to the reversible silent state of the HIV-1 provirus in cells and tissues called viral reservoirs [21]. Latency of the HIV-1 provirus is a significant component of the pathogenesis of the retrovirus [22–24].

Epigenetic and epitranscriptomic regulation encompasses chemical modifications to nucleic acids and the proteins with which they associate in order to effect gene expression without altering the DNA sequence. These modifications determine the conformation of chromatin and whether it exists in an open, transcriptionally active state or a compact, repressive state [25]. Because retroviruses integrate into the host genome, they are subject to the same epigenetic and epitranscriptomic controls that govern host genes. The balance between activation and repression of integrated viral DNA and its RNA products defines retroviral latency and persistence, providing the foundation for the mechanisms discussed below.

**Fig. 1:**
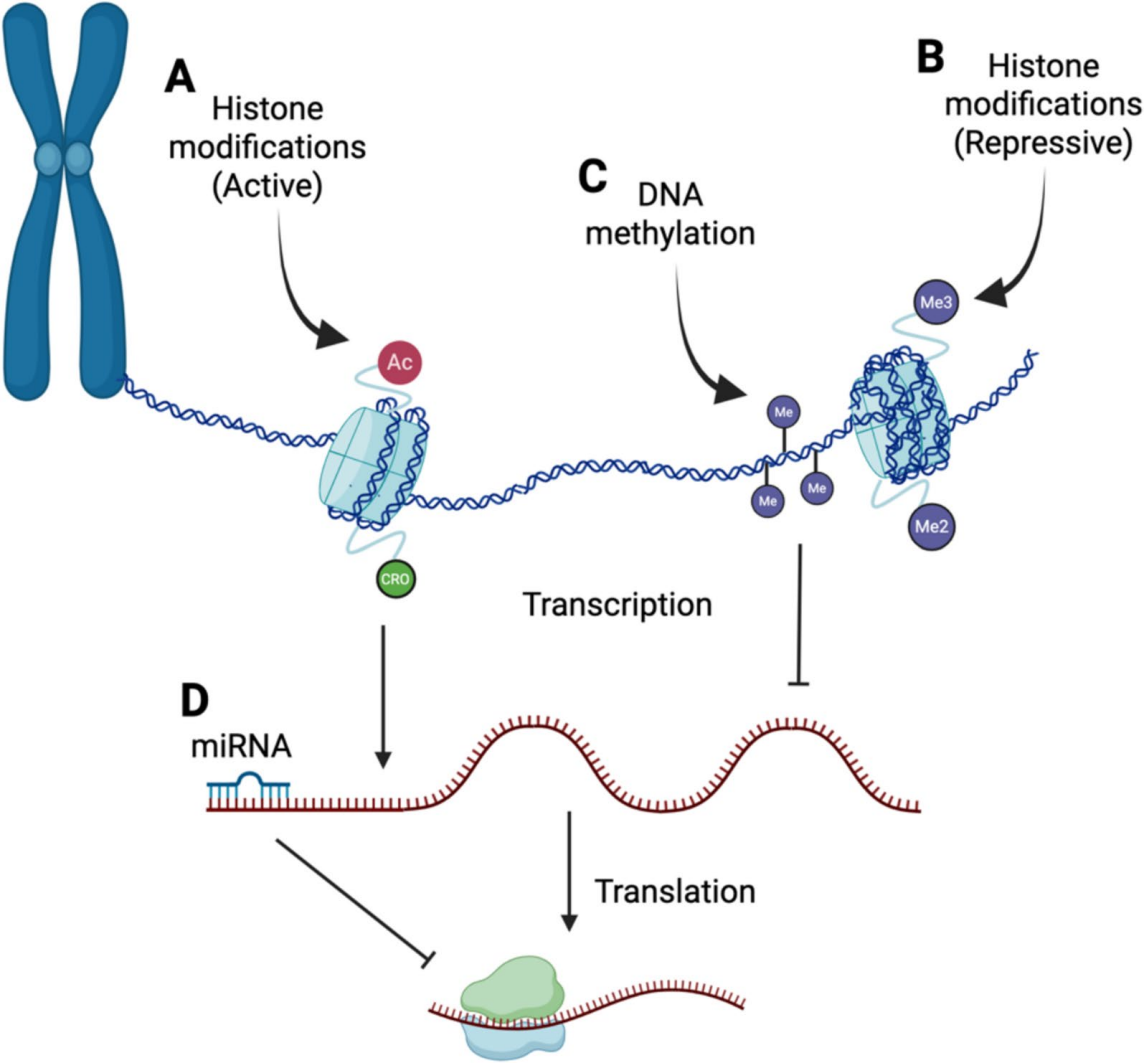
Epigenetics overview. Histone modifications can accompany both 'active' (**A**) and 'repressed' (**B**) chromatin, while DNA methylation (**C**) is repressive when located in gene promoters. Post-transcriptional regulation through microRNAs (miRNA) and other non-coding RNAs (**D**) is another epigenetic mechanism regulating gene expression. Image generated with www.biorender.com

Early studies proposed that transcriptional repression through DNA-binding proteins, chromatin conformation, and DNA hypermethylation bolstered HIV-1 latency [26–29]. Owing to the extensive research into HIV-1 from the last few decades, epigenetic silencing mechanisms for HIV-1 are better-characterized than for any other retrovirus, and perhaps all other viruses in general. However, our understanding of the processes guiding HIV-1 latency in multiple primary cell types with variable tissue environments is still relatively poor, as many of the mechanisms defined in cell lines and even primary cells isolated from peripheral blood do not necessarily carry over to all of these scenarios. The main modes of epigenetic and epitranscriptomic control include those provided by DNA methylation, histone modifications, and non-coding RNAs (Fig. 1).

### DNA methylation of integrated HIV-1 DNA

HIV proviral DNA integrates into the host genome and makes use of its cellular machinery for transcription. This process has the potential to make the viral genome susceptible to genetic silencing through DNA methylation [30]. DNA methylation, a pre-transcriptional epigenetic mechanism, utilizes DNA methyltransferases (DNMTs) to transfer a methyl group to the fifth carbon of cytosine residues (5-methylcytosine, or 5mC), typically in palindromic CpG dinucleotides [30]. DNA methylation often results in inhibition of transcription factor binding to their recognition sites, and recruiting chromatin remodeling co-repressor complexes to repress transcription [31]. Regions of the DNA sequence densely populated with CpG dinucleotides or residues are termed as CpG Islands (CGIs) and are associated with gene repression when hypermethylated [21]. CpG methylation around the 5’ long terminal repeat (LTR) CGIs and non-coding leader region CGIs around the transcription start site of the HIV-1 promoter have been examined to provide a better understanding of HIV-1 latency [21]. An *in vitro* study conducted by Kauder et al*.* showed that in Jurkat cells and latently infected CD4 + T cells, two CGIs near the promoter were hypermethylated [32]. The potential for a repressive chromatin state as the regulator of latency mediated by promoter methylation was further supported by the presence of methyl-CpG binding domain protein 2 (MBD2) and histone deacetylase 2 (HDAC2) [32]. While chromatin modifying enzymes such as HDACs have long been established regulators of HIV-1 latency [33], emerging evidence shows that MBD2 causes transcriptional repression by inciting nucleosome remodeling and recruiting the histone deacetylation (NuRD) complex, which provides another level of chromatin remodeling [32].

DNA methylation has been primarily studied around the 5' LTR, which contains promoter and enhancer regions, causing transcriptional suppression of the provirus [34]. This potentially contributes to the ability of HIV-1 to escape host immune detection and antiretroviral drugs, allowing for the establishment of latency throughout tissue compartments in the body. A study by Bednarik et al*.* identified methylation of two CpG sites responsible for silencing reporter genes and infectious proviral DNA in HIV-1, which was countered using the trans-activator Tat [26]. This result implied that methylation of the LTR could alter the transcriptional properties of the HIV-1 LTR region to potentially allow for latency.

DNA methylation in CpG sites is rarely studied in people with HIV (PWH), particularly those on ART. ART has been utilized as the clinical management plan for PWH in the past few decades, transforming HIV from a fatal disease to a chronic one by inhibiting HIV replication. Despite ART, the HIV provirus persists in viral reservoirs throughout the body in PWH, allowing the virus to evade the immune system until it can be reactivated [21]. The role of proviral DNA methylation in HIV latency and persistence has remained unclear, as inconsistent results are reported in clinical samples. Blazkova et al*.* reported that in the latent reservoir of aviremic PWH, the 5’LTR is hypermethylated and resistant to reactivation in CD4 + T cells [34]. Conversely, a later study by the same group concluded that the latent reservoir of aviremic PWH on ART had a scarcity of DNA methylation in the 5’LTR [35]. Consistent with this latter result, Trejbalova et al. also reported PWH on ART exhibited little DNA methylation, which seemed to increase with longer exposure to ART [36]. Another study compared the percentage of methylation of the 5’LTR in aviremic PWH on ART, long-term nonprogressors, and elite controllers, finding that PWH on ART had a significantly lower methylation percentage [37]. Further, Palacios et al*.* found that the 5' LTR was hypermethylated with an increasing duration of infection [37]. These studies associated increased DNA methylation in the 5’LTR with duration of infection, which is further supported by a longitudinal study with PWH on ART for twelve months [38]. Though this study tries its best to address the lack of evidence for DNA methylation in clinical samples from people on long-term suppressive ART, it does not go into depth about the heterogeneity in PWH. For example, while some PWH developed a significant increase in methylation, others exhibited continuously elevated methylation for the duration of the study [38]. Lastly, Weber et al*.* concluded that DNA methylation does not play a primary role in regulating HIV-1 transcription after examining DNA methylation in peripheral blood mononuclear cells (PBMCs) from twenty-three PWH [39]. The variability in conclusions from these studies could be attributed to heterogeneity of retroviral infections in general and/or fluctuations in proviral clones able to be sampled in peripheral blood; however further studies are required to tackle the challenge of elucidating the full explanation for the role of HIV proviral DNA methylation.

The three DNMTs, DNMT1, DNMT3a, and DNMT3b, play a crucial role in determining the methylation of cytosines in both CpG and non-CpG residues [40]. DNMT1, also called the “maintenance” methyltransferase, targets hemimethylated DNA, making CpG methylation prevalent in dividing cells [41]. However, the lack of DNMT1 in nondividing cells such as neurons and ESCs releases the restriction of cytosine methylation to CpG residues and allows for DNA methylation in CHG and CHH contexts (H = A, G, or T) [42]. Cells with non-CpG methylation have a higher expression of the *de novo* methyltransferases DNMT3a and DNMT3b [42]. Yan and colleagues demonstrated that whole genome bisulfite sequencing of the transcription factor A, mitochondrial (TFAM) promoter in human skeletal muscle showed 3.2% CpA, 2.3% CpT, and 1.6% CpC methylation compared to unmethylated controls, establishing the existence of non-CpG methylation [42]. Though it has been reported in the literature that cytosine methylation exists in the non-CpG residues in mammalian DNA [40], it has not been an extensively researched topic in the context of retroviruses. Non-CpG methylation has been reported in exogenous MLV infection through the use of bisulfite analysis in the humanized green fluorescent protein (hGFP) gene [43]. Another study conducted by Dodge et al*.* confirms that the presence of *de novo* DNMT3 enzymes is correlated with non-CpG methylation in CpA residues in MLV [44], suggesting that non-CpG methylation likely signifies the presence of *de novo* DNA methylation and does not necessarily carry functional consequences independent of the more prevalent CpG methylation.

Importantly, the apparent lack of functional relevance attributed to proviral DNA methylation in HIV-1 has largely been inferred from PCR-based assays, which are inherently insensitive to methylated templates, particularly when non-CpG methylation is abundant. As a result, non-CpG methylation may contribute to systematic under-detection of proviral DNA methylation, rather than reflecting a lack of biological relevance. Early studies by Fang et al*.* and Mikovits et al*.* demonstrated that HIV-1 infection of CD4 + T cells induces DNMT expression and *de novo* DNA methylation, including in the context of integration-defective virus [45, 46], and intracellular Tat has since been shown to induce genome-wide changes in DNA methylation [47]. Consistent with these findings, whole-genome bisulfite sequencing revealed substantial non-CpG methylation across the proximal HIV-1 provirus in PBMCs from PWH [48]. In contrast, sequence-specific PCR-based approaches, particularly those relying on nested amplification, are prone to bias toward unmethylated templates due to primer design constraints and stochastic amplification effects. Together, these findings suggest that proviral DNA methylation in HIV-1 has likely been underestimated in clinical samples and highlight the need for methylation-agnostic approaches to more accurately define the functional role of DNA methylation in retroviral infections.

### Histone modifications

Nucleosomes are the basic packaging unit of chromatin and are composed of a histone octamer, which includes two copies of each histone (H3, H4, H2A, and H2B). DNA is wrapped around these octamers in approximately 147 base pair increments. Chromatin is organized into tightly packed and inaccessible regions of DNA, termed heterochromatin, and open and accessible regions of DNA termed euchromatin. Eukaryotic cells use multiple methods to regulate this packaging, including histone variants, ATP-dependent chromatin remodelers, histone chaperones that regulate the movement of histones, and histone modifications [49].

**Fig. 2:**
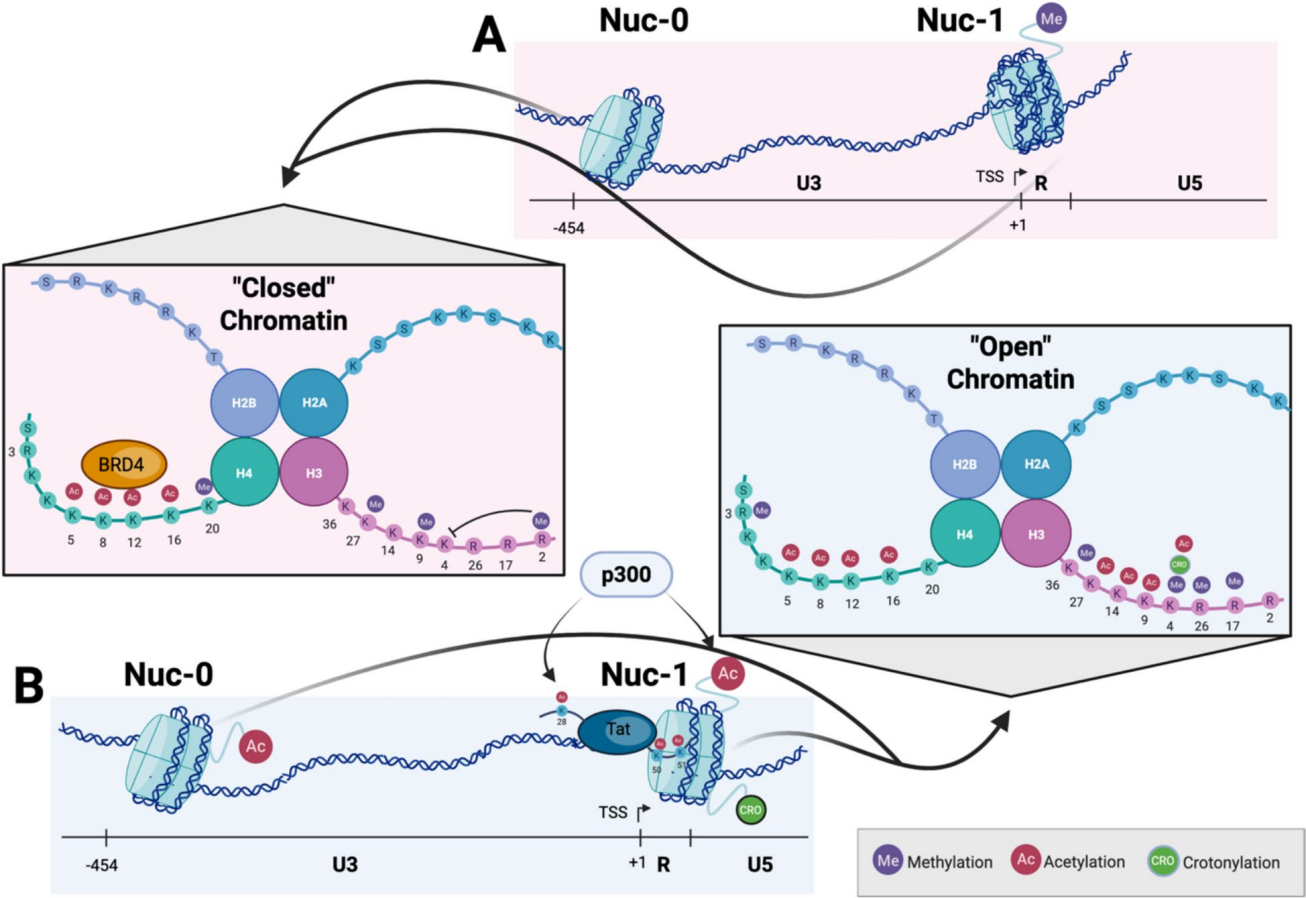
Histone modifications in the HIV-1 LTR. **A** During HIV-1 latency, 'closed' chromatin with Nuc-1 blocking the transcriptional start site (TSS) is maintained by histone deacetylases, histone methylation on H3K27, H3K9, and H3R2, and H4K20. BRD4 is also recruited to acetylated lysine tails on H4. **B** During HIV-1 transcriptional activation, chromatin relaxation and Nuc-1 displacement occurs as a result of SWI/SNF recruitment due to Tat accumulation and acetylation by p300, allowing access to the transcriptional start site (TSS). Histone acetyltransferases (e.g. p300) acetylate both Tat and histones H3 and H4, while histone demethylases and methyltransferases modify histones for a more 'active' conformation, including methylation of H3K36, H3K4, H3R26, and H3R17 and acetylation of multiple lysine tails on H3 and H4, as well as crotonylation on H3K4. Image generated with www.biorender.com

Over 200 histone modifications have been described, leading to permutations of 3000 to 4000 combinations that regulate chromatin compaction [50, 51]. Of these, at least nine major variants have been characterized, including lysine acetylation, lysine and arginine methylation, phosphorylation, ubiquitylation, ADP ribosylation, lysine SUMOylation (i.e. addition of small ubiquitin-like modifier), and lysine crotonylation. These modifications are typically located on the N- or C-terminal tails of histones, although some have been reported in the globular domains of histones as well [52]. Histone modifications themselves not only alter DNA compaction, but they also frequently serve as docking sites for multiple regulators of gene expression and chromatin compaction. These modifications and their regulators work in concert to direct chromatin organization and undertake gene regulation [49, 53]. In addition to covalent histone modifications, nucleosome positioning itself contributes to latency. The SWitch/Sucrose Non-Fermentable (SWI/SNF) subcomplex BRG1/BRM-Associated Factor (BAF) actively positions the repressive Nuc-1 nucleosome over the transcription start site, a configuration required to maintain proviral silencing [54], while polybromo-associated BAF (PBAF) subcomplexes are required for chromatin remodeling and activation at Nuc-1 [55]. Several of the most commonly studied histone modifications examined in the context of HIV-1 proviral control are outlined in Fig. 2 and Table 2.

**Table 2:** Histone modifications and their writers/erasers reported in the HIV provirus

Histone modification	Activation-associated	Writers	Erasers	Repression-associated	Writers	Erasers	Context-dependent	Writers	Erasers
Histone lysine acetylation	H3K9ac	GCN5, P/CAF, CBP/P300	HDAC1/2/3	N/A			H4K5ac	KAT5, KAT7, CBP/P300	HDAC1/2/3
	H3K14ac	KAT5, KAT7, GCN5, P/CAF, CBP/P300	HDAC1/2/3				H4K8ac	KAT5, CBP/P300	HDAC1/2/3
	H3K27ac	CBP/P300	HDAC1/2/3				H4K12ac	KAT5, KAT7, CBP/P300	HDAC1/2/3
							H4K16ac	KAT5, KAT8, CBP/P300	SIRT1, HDAC1/2/3
Histone lysine methylation	H3K4me3	SMYD2, SET1A, WDR/MLL	KDM5A	H3K27me2/3	EZH2 (PRC2)	UTX-1	H4K20me1	SMYD2	Unknown
	H3K36me3	SETD2, SMYD2	MINA53	H3K9me2/3	EHMT1 (GLP), EHMT2 (G9a), SETDB1, SETDB2, SUV39H1	LSD1 (H3K9me2 only)			
	H3K4me1	SETD7, SET1A, WDR/MLL	LSD1	H4K20me3	SUV4-20H, SMYD5	Unknown			
	H3K4me2	SET1A, WDR/MLL	LSD1						
Less-studied histone modifications	H3R17me2	CARM1/PRMT4	Unknown	H3R2me2a	PRMT6	Unknown	H3R26me2	CARM1/PRMT4	Unknown
	H4R3me2a	PRMT1	Unknown	H2AK119ub	RING1 (PRC1)	Unknown in HIV			
	H3K4cr	ACSS2	HDAC1/2/3, SIRT1	H4 SUMOylation	SMC5/6	Unknown in HIV			

#### Histone lysine acetylation

Histone lysine acetylation is one of the most commonly studied histone modifications regulating integrated HIV-1 DNA, with the major focus being on modifications of the three nucleosomes in the proximal provirus (Nuc-0, Nuc-1, and Nuc-2). One of the earliest events at the viral LTR upon reactivation is the recruitment of histone acetyltransferases (HATs), including CREB-binding protein (CBP), general control of amino acid synthesis protein 5-like (GCN5), and P300/CBP Associated Factor (P/CAF), also called lysine acetyltransferase 2B (KAT2B). CBP recruitment to the LTR in particular is Tat-independent and works as a transcriptional coactivator of NF-kB with the HAT p300, indicating NF-kB binding likely recruits the initial factors needed for proviral chromatin remodeling. Recruitment of HATs results in increased global H3 and H4 acetylation of all three proximal nucleosomes in the provirus and also frequently modulates Tat function via acetylation of Tat itself. Specific acetylation marks described in the context of HIV-1 include H3K9ac, H3K14ac, H3K27ac, H4K5ac, H4K8ac, H4K12ac, and H4K16ac [56–58]. H3K27 acetylation in particular was shown to recruit the super elongation complex (SEC), which is essential for HIV-1 LTR-directed transcription. Additionally, H3K27ac stimulates H3R26 methylation, which ameliorates SEC recruitment and forms a negative feedback loop [57].

HDACs, which remove acetylation from histones, serve to silence the provirus, and inhibitors of these enzymes have repeatedly been shown to reverse latency to varying degrees [56, 59]. HDAC1 was first found in a complex with transcriptional repressors ying-yang factor 1 (YY1) and the late simian virus 40 factor (LSF) at the LTR [27]. There are currently eighteen known HDAC proteins belonging to four classes based on sequence similarity, with the majority of those linked to LTR deacetylation belonging to Class I [56]. The host factor COUP-TF interacting protein (CTIP2) recruits histone deacetylases (HDACs) HDAC1 and HDAC2 to the 5' LTR of the provirus in monocytes and macrophages [60] and in CD4 + T cells [61] to maintain silencing. Because of their key role in latency, HDACs have received scrutiny for their role in HIV-1 latency reversal for many years [62, 63]. Recent work in a primary CD4 + T cell latency model has suggested that HDAC1/2 and HDAC3 must all be targeted to prevent latency and/or achieve latency reversal [64].

Histone lysine acetylation is most frequently associated with active transcription, although in the context of HIV-1, H4 acetylation can lead to a recruitment of the bromodomain and extraterminal motif (BET) protein BRD4 (bromodomain containing 4), which is known to silence the provirus via competition with Tat binding to positive transcription elongation factor b (P-TEFb) [65, 66]. Earlier work demonstrated that the BAF complex establishes the repressive Nuc-1 configuration at the LTR [54], providing a mechanistic foundation for subsequent studies linking BRD4 isoforms to SWI/SNF-mediated repression. The enzyme lysine acetyltransferase 5 (KAT5) acetylates H4 in order to recruit BRD4 and promote latency in T cells [58]. However, the picture appears increasingly complicated, as modulation of BRD4 can either activate or suppress proviral transcription, depending on the partner proteins of BRD4 [67]. The outcome is dependent on whether inhibition of BRD4 disengages it from H4 acetylation, which will release cyclin-dependent kinase 9 (CDK9) and, in turn, promote CDK9-Tat binding. Conversely, modulation of BRD4 that maintains its association with H4ac while promoting CDK9-BRD4 binding will inhibit CDK9-Tat association and disrupt HIV-1 transcription [67]. BRD9, another member of the BET protein family, has also been shown to interfere with Tat binding at the HIV-1 LTR, leading to a repressive chromatin environment, and repression of this protein alongside that of BRD4 acts in a synergistic manner to cause latency reactivation in CD4 + T cells [68]. Whether BRD9 is capable of dual activity as with BRD4 has not yet been addressed in the literature.

#### Histone lysine methylation

Histone lysine and arginine residues can be mono-, di-, or trimethylated, and the specific residues modified will associate them either with transcriptional activation or repression. Among the most commonly studied histone methylation marks are H3K4me, H3K9me, and H3K27me, both in the context of HIV-1, and in general. Increasing methylation on these residues often maximizes their associated effects, e.g. H3K27me3 is maximally associated with repression vs. H3K27me2. Further complicating the picture, many histone modifying enzymes also modify Tat itself, resulting in modulation of the enzyme [69].

H3K27me3 in the LTR has been repeatedly shown to be important for HIV-1 silencing. The H3K27 methyltransferase Enhancer of Zeste Homolog 2 (EZH2), which is part of the Polycomb Repressive Complex 2 (PRC2), associates with the promoter-enhancer region of HIV-1 in latently infected Jurkat cell lines. Knockdown of EZH2 reactivates a significant portion of latent proviruses and results in sensitization to external stimuli, preventing reversion to latency [70, 71]. Chemical inhibition of EZH2 with GSK-343 reduced H3K27me3 in the LTR of the latent provirus without concomitant reactivation in CD4 + T cells, but sensitized the provirus to reactivation with the HDAC inhibitor Vorinostat, indicating methyltransferase inhibitors could synergize with HDAC inhibitors to promote proviral reactivation [72]. However, chemical inhibition of EZH2 with either GSK-343 or EPZ-6438 in a Th17 primary cell latency model and in memory CD4 + T cells isolated from aviremic PWH demonstrated reactivation of latent proviruses, suggesting the picture is more complicated [72]. In Jurkat cells, knockout of EZH2 resulted in depletion of both EZH2 and the H3K9 methyltransferase Euchromatic Histone lysine Methyltransferase 2 (EHMT2) in the HIV-1 promoter, resulting in reactivation. Knockout of EHMT2 alone failed to reactivate the provirus, suggesting that H3K27me3 modulation is a critical first step in reversing latency. Likewise, both PRC2 and G9a (the H3K9 methyltransferase complex to which EHMT2 belongs) are enriched at the LTR and rapidly displaced upon reactivation [70].

Ubiquitously Transcribed X-chromosome 1 (UTX-1), one of the two histone demethylases responsible for H3K27 demethylation, was demonstrated to be required for Tat removal of H3K27me3 in the LTR in TZM-bl cells (a HeLa cell line permissive to HIV infection). It was also shown to promote HIV-1 expression by facilitating Nuclear Factor Kappa B (NF-kB) p65 translocation to the nucleus. Tat dramatically increased UTX-1 transcription and protein levels, while knocking down UTX-1 in the presence of Tat resulted in a rescue of H3K27me2/3 [73]. More recent work demonstrated that knocking down UTX resulted in increased H3K27me3 at the HIV-1 promoter in CD4 + T cell lines. This increase was associated with a block to reactivation and with a recruitment of DNMT3a and a subsequent increase in DNA methylation at the promoter, resulting in a more permanent silencing mechanism. Conversely, proviral reactivation was associated with an increase in UTX and a decrease in EZH2 and H3K27me3 in the proximal provirus [74]. Recent work has identified Schlafen-5 as a key player in these pathways, suggesting it inhibits HIV-1 replication by binding to the 5' LTR and recruiting the PRC2 and G9a complexes while interacting directly with H3, ultimately promoting H3K9me2/3 and H3K27me2/3 deposition [75].

H3K9me2/3 are also frequently associated with heterochromatization and subsequent HIV-1 latency. Histone methyltransferases EHMT1 (GLP) and EHMT2 (G9a) catalyze dimethylation of H3K9 in euchromatic regions [76]. Aside from dimerizing and participating in a couple of other documented histone modifications, these Suppressor of Variegation 39 Homolog (SUV39H) family members also methylated non-histone proteins, including CCAAT Enhancer Binding Protein beta (CEBPb), DNMT1, HDAC1, Krüppel-Like Factor 12 (KLF12), and Tumor Protein 53 (P53) [76]. Both proteins dimethylated H3K9 in the HIV-1 LTR to promote repression [77]. GLP knockdown resulted in HIV-1 LTR reactivation in cell lines [78], while chemical inhibition of G9a by UNC0638 reactivated proviruses in a Th17 primary cell latency model [70]. SET Domain Bifurcated 1 (SETDB1 or ESET), another H3K9 methyltransferase, regulates H3K9me3 in endogenous and introduced retroviruses [79]. This enzyme also associates with HIV-1 Tat and methylates it at lysines 50 and 51. Therefore, its knockdown resulted in decreased HIV-1 transcription despite its association with heterochromatization. SETDB2, another H3K9 methyltransferase responsible for H3K9me3 accumulation, was also associated with Tat in co-IP experiments, although its function is less clear [80]. Suppression of variegation 3–9 homolog 1 (SUV39H1) produces H3K9me3 using H3K9me1 as a substrate. SUV39H1 catalytic activity is reduced by H3K4me3, and it complexes with other H3K9 methyltransferases, including GLP, G9a, and SETDB1, creating a binding site for HP1a, which binds H3K9me2/3. In turn, Heterochromatin Protein 1-alpha (HP1a) recruits SUV4-20H enzymes to heterochromatic regions to generate H4K20me3 [69]. One group found that SUV39H1 and HP1a are associated with increased H3K9me3 and repression of integrated HIV-1 in several systems, including peripheral blood mononuclear cells (PBMCs) from PWH [81]. In microglial cells, CTIP2 bound to DNA associated with SUV39H1, increasing H3K9me3 at the HIV-1 LTR and recruiting HP1 proteins to promote heterochromatin formation [61]. Induction of HIV-1 expression was also associated with loss of H3K9me3 and gain of H3K9ac at the HIV-1 promoter. Treatment with chaetocin, an SUV39H1/G9a inhibitor, caused a 25-fold induction of latent provirus in Jurkat cells [82].

A clustered regularly interspaced short palindromic repeats (CRISPR) / CRISPR-associated protein 9 (Cas9) screen identified Zinc Finger 304 (ZNF304) as a mediator of latency, and further investigation revealed that it recruits Tripartite Motif Containing 28 (TRIM28/KAP1) and the chromatin modifiers PRC1 and 2, as well as SETDB1 to the LTR. Consequently, depletion of ZNF304 resulted in an increase in H3K27me3 and H3K9me3, as well as H2AK119ub, which is essential for polycomb-mediated transcriptional repression [83]. A study of Nerve Growth factor 1B-like receptor (Nurr1) in microglial cells also found that this protein binds directly to the HIV-1 LTR U3 region to ameliorate expression via recruitment of CoREST/HDAC1/G9a/EZH2 complexes to the LTR [84]. Additionally, RING1 and YY1 binding protein (RYBP), a H2AK119ub reader, was recently reported to promote HIV-1 latency by binding to YY1 at the HIV-1 LTR, culminating in increased ubiquitination of H2AK119 and depletion of H3K4me3 [85].

The HIV-1 provirus is additionally impacted by histone methylation marks canonically associated with transcriptional activation. H3K36me3 plays an important role throughout the viral life cycle, as it is critical for initial tethering of integrase to chromatin via Lens Epithelium Derived Growth Factor (LEDGF/p75), a nuclear protein that serves as a docking factor for pre-integration complexes and is a reader for H3K36me3 [86, 87]. This mark is typically found throughout gene bodies of actively transcribed genes. SET Domain Containing 2 (SETD2) trimethylates H3K36 and is recruited to RNA Pol II at the HIV-1 LTR in HLM107 cells, a HeLa cell line infected with a single Rev-defective HIV-1 provirus. This enzyme is required for H3K36me3 across the transcribed region of the provirus in this system [88]. A recent CRISPR/Cas9 screen in JLAT cells found that the histone demethylase, Myc Induced Nuclear Antigen 53 (MINA53), promoted latency. MINA53 preferentially demethylates H3K36me3, and its deletion with RNA interference (RNAi) resulted in increased H3K36me3 at the HIV-1 LTR. KAT8, a reader of H3K36me3, was also found through shRNA experiments to recognize H3K36me3 at the LTR and increase H4K16ac [89].

Additionally, the lysine methyltransferase Su(var)3–9, enhancer-of-zeste, and trithorax (SET) and myeloid, nervy, and DEAF-1 (MYND) domain-containing protein 2 (SMYD2) was discovered as a latency factor in an RNAi screen of human lysine methyltransferases in JLAT cells. SMYD2 ordinarily methylates H3K36 and H3K4 [90, 91]. However, in the context of latent HIV-1 in JLAT cells, it appeared to methylate H4K20me1 more heavily. Knockdown of SMYD2 and also its inhibition with AZ391 was found to reverse latency in JLAT cells, and chromatin immunoprecipitation (ChIP) demonstrated the association of SMYD2 with the LTR in this latency model. Methylation of H4K20 by SMYD2 recruited Lethal Malignant Brain Tumor-Like protein 1 (L3MBTL1), which serves to promote latency [76]. More recent work has established SMYD5 as a requirement for HIV-1 transcription, as it methylates Tat in CD4 + T cells *in vitro* [92]. Interestingly, SMYD5 is ordinarily responsible for H4K20me3 maintenance at Long Interspersed Nuclear Elements (LINEs) and repetitive elements in murine ESCs [93], instead highlighting it as a repressive mechanism for endogenous elements during differentiation.

SETD7, which monomethylates H3K4, also coactivates HIV-1 transcription. In JLAT cells it associates with the HIV-1 promoter, binds Trans-Activation Response (TAR) RNA to form a complex with P-TEFb and Tat, and methylates Tat at lysines 51 and 71, suggesting it has a role in early Tat transactivation [94, 95]. Lysine-Specific histone Demethylase 1 (LSD1), a histone lysine demethylase, demethylates H3K4me1 and H3K4me2. This enzyme associates with the HIV-1 promoter along with the CoREST complex in order to activate Tat in T cells. In turn, it is also a Tat-specific demethylase at K51 [96]. LSD1 cooperates with CTIP2 in silencing the HIV-1 provirus in microglial cells by reducing H3 acetylation. It is associated with both increasing H3K4me3 and H3K9me3. LSD-induced H3K4me3 deposition appears to recruit the H3K4 methyltransferase SET1A (hSET1) to the provirus, providing a feed-forward mechanism to increase H3K4me3. LSD1 also interacts with H3K9 demethylases and HDAC1 and HDAC2, suggesting that it can play a role in both transcriptional activation and repression [97]. Conversely, Liu et al*.* reported that LSD1 enhances Tat-induced transcriptional activity in HeLa-based NH1 cells [98], consistent with what Sakane et al. reported [96]. In their model, LSD1 promoted HIV-1 transcription and was associated with a reduction of H3K4me3 at Nuc-1. LSD1 is not directly capable of H3K4me3 demethylation, but it was found to recruit KDM5A, another H3K4 demethylase, and to inhibit the H3K4 methyltransferases SET1A and WDR binding at the LTR [98].

Finally, a recent study by Lu et al*.* suggests that placement of these marks is important and variable between cell types and genomic contexts, and their associated transcriptional effects are also variable as a result. They found that HIV-1 genomes in monocyte-derived macrophages exhibited active transcription despite extensive H3K9me3 across the genome, although H3K9ac and H3K27ac were accumulated at the LTR. In contrast, JLAT cells exhibited bivalent H3K4me3 and H3K27me3 accumulation at the LTR, while primary CD4 + T cells had intermediate H3K9me2/me3 across the provirus, lower H3K4me3 and H3K27me3, and no specific enrichment at the LTR [99]. This study provides an important reminder that the transcriptional profiles associated with specific histone modifications are combinatorial and context-dependent, demonstrating that no single histone modification at specific loci should be interpreted in isolation.

#### Less-studied histone modifications

Some other less-studied histone modifications have also been described in the context of HIV-1. Arginine methylation is one such modification. Coactivator of arginine methyltransferase 1 (CARM1 or PRMT4) is an arginine methyltransferase responsible for asymmetrical H3R17 and H3R26 dimethylation and is recruited to promoters during active gene transcription, methylating many transcription factors in its wake. It interacts with H3K27ac and is associated with active gene transcription [57]. PRMT1 asymmetrically methylates H4R3me2a and is associated with NF-kB-mediated expression at the HIV-1 promoter [100]. Protein Arginine Methyltransferase 6 (PRMT6) catalyzes dimethylation of H3R2me2a and serves as a negative regulator of H3K4me3 deposition by blocking recruitment of the H3K4 methyltransferases WD Repeat Domain 5 (WDR5) and Mixed Lineage Leukemia (MLL), thus associating with decreased HIV-1 transcription. This enzyme was also shown to methylate Tat, restricting viral transcription and replication [101]. Histone crotonylation is also a regulator of HIV-1 latency. Jiang et al. reported that increased expression of Acyl-CoA synthetase short chain family member 2 (ACSS2) resulted in histone crotonylation and reactivation of HIV-1 in primary CD4 + T cells [102].

### Non-coding RNAs

Non-coding RNAs (ncRNAs) are RNA molecules that do not encode proteins, but instead regulate gene expression at transcriptional and post-transcriptional levels. Multiple cellular non-coding RNAs (ncRNAs) provide another layer of epigenetic and epitranscriptomic regulation to retroviruses, which have been heavily documented in HIV-1. These include microRNAs (miRNAs), piwi RNAs (piRNAs), and long non-coding RNAs (lncRNAs). Additionally, ncRNAs are also generated directly from viral sequence through multiple mechanisms.

Primary miRNA transcripts are processed in the nucleus by the endonuclease DROSHA into 70–100 bp-long pre-miRNAs. Pre-miRNAs are exported into the cytoplasm and further processed by the ribonuclease DICER to generate ~ 22 bp long miRNAs. MiRNAs are predominantly involved in post-transcriptional gene expression regulation in the cytoplasm, where the RNA-induced silencing complex (RISC) guides them to specific mRNAs to downregulate gene expression through miRNA-mediated degradation [103] or translational repression [104]. By downregulating expression of transcription factors in this manner, miRNAs indirectly act in epigenetic regulation of gene expression. Additionally, miRNAs can also enter the nucleus to induce [105, 106] or repress [107] gene transcription.

In the context of HIV, miRNAs can impact regulators of HIV-1 or HIV-1 RNA directly. Several miRNAs have been described that target activators of HIV-1, leading to latency. These include miR-155, which targets tripartite motif containing 32 (TRIM32) to promote HIV-1 latency in J-LAT 5A8 cells, a CD4 + lymphoblastic T cell line [108], as well as miR-186 and miR-210, which target HIVEP zinc finger 2 (HIVEP2) in the lymphoblastic lymphoma line SUP-T1 [109]. Several miRNAs were reported to interact with Tat cofactors to promote latency, including miR-27b, miR-29b, miR-150, and miR223 [110]. miR-198 was reported to target cyclin T1 in Mono Mac cells, which are a promonocytic cell line [111], while miR-17-5p and miR-20a caused translational inhibition of P/CAF (or KAT2B) in the Jurkat lymphoblastic cell line, the U1 promonocytic cell line, and PBMCs from PWH, inhibiting viral replication [112].

Other miRNAs target viral repressors, resulting in reactivation of HIV-1 from latency. Examples include miR-9-5p, which negatively regulates PR/SET domain 1 (PRDM1) in HUT78 cells, a T cell lymphoma line [113]. PRDM1 ordinarily binds the HIV-1 LTR to recruit HDACs [114] and inhibit transcription [115]. Silencing miR-9-5p in primary CD4 + T cells increased PRDM1 expression and was associated with the establishment of latency [116]. Knockout of miR-146a with CRISPR-Cas9 in infected leukemic MT2 cells led to the upregulation of HIV-1 restriction factors and downregulation of HIV-1 expression; its abrogation in Jurkat cells infected with a replication incompetent virus resulted in suppression of reactivation by the HDAC inhibitor suberoylanilide hydroxamic acid (SAHA) [117]. MiR-139-5p targets Forkhead Box O1 (FOXO1) [118], which restricts HIV-1 [119], and experimental upregulation of this miRNA resulted in increased viral reactivation [118].

MiRNAs derived from the HIV-1 genome have also been described. TAR-derived miRNAs, documented in both CD4 + T cells [120] and macrophages [121] can promote RISC inhibition and stunt recruitment of HDAC1 to the HIV-1 5' LTR, creating an environment that supports latency [122, 123]. Additionally, hiv1-miR-H1 can induce cellular responses that both promote and antagonize latency [124], as it interacts with both promoters and repressors of HIV-1 transcription [125, 126].

piRNAs are 26–31 nucleotide long ncRNAs that are associated with PIWI proteins, which are defined by the presence of the PIWI-AGO-Zwille (PAZ) domain responsible for RNA binding, and modified with 2'-0-methyl at their 3' termini. piRNAs are typically generated from transposons, and in the past were considered only germline expressed [127]. Their generation and function are coupled by a "ping-pong" cycle in germ cells [128]. However, increasing evidence suggests that piRNAs or piRNA-like molecules are widely expressed in human somatic cells and cancer cells. piRNAs or piRNA-like molecules that are associated with PIWI proteins not only silence retrotransposons in germ cells, but also regulate the expression of various genes in somatic cells under certain physiologic and pathologic processes [114, 129–132]. piRNAs can regulate expression through several mechanisms, including at the transcriptional level by inducing DNA methylation and histone modifications, resulting in heterochromatin formation at target loci, but also by mRNA degradation at the posttranscriptional level.

In HIV literature, two PIWI proteins have been described in transcriptional regulation, but no specific piRNAs. Knockdown of the PIWI protein PIWIL4 in latently infected Jurkat cells and primary CD4 + T cells resulted in HIV-1 reactivation. PIWIL4 interacts directly with the 5' HIV-1 LTR and suppresses its expression through recruitment of repressive histone modifiers. However, this process appeared piRNA-independent [133]. PIWIL2 inhibits active HIV-1 replication in activated CD4 + T cells by hijacking rare tRNA species needed for HIV-1-encoded proteins [134], but this process does not appear related to latency.

LncRNAs are non-coding transcripts > 200 bp that regulate expression either transcriptionally or post-transcriptionally through secondary structures that (1) serve as scaffolds for protein complexes; (2) guide proteins to their targets; (3) serve as molecular ‘sponges’ for proteins and regulatory miRNAs [135, 136]. As scaffolds for protein complexes and guides, lncRNAs may directly participate in epigenetic regulation; while ‘sponging’ proteins and miRNAs results in indirect regulation (e.g., changing the availability of transcription factors for promoter binding).

In the context of HIV, lncRNAs act to promote or reverse HIV-1 latency through epigenetic mechanisms. For example, inhibition of the lncRNA *AK130181* results in viral reactivation in latently infected Jurkat and primary CD4 + T cells, as well as resting CD4 + T cells from people on ART, suggesting it promotes latency through epigenetic mechanisms [137]. NFkB-interacting lncRNA (*NKILA*) inhibits replication of multiple HIV subtypes in HEK293 [138]. Both of these lncRNAs appear to act in an NFkB-dependent manner [138, 139]. Plasmacytoma variant translocation 1 (*PVT1*) is upregulated in two T cell models of HIV-1 latency [139] and increases expression of the H3K27 methyltransferase EZH2, a transcriptional repressor implicated in HIV-1 latency [70, 72], in hepatocellular carcinoma [140].

Experimental reduction of metastasis-associated lung adenocarcinoma transcript 1 (*MALAT1*) expression led to decreased viral transcription and replication [141], suggesting that this lncRNA acts as an HIV-1 activator. It associates with PRC2 to inhibit EZH2 binding to the HIV-1 promoter, resulting in inhibition of H3K27 methylation [141]. HIV enhanced lncRNA (*HEAL*) is associated with the RNA-binding protein, Fused in Sarcoma (FUS), to promote viral replication through 2 mechanisms: (1) binding the promoter to recruit HAT p300, resulting in increased H3K27 acetylation and p-TEFb recruitment; and (2) enhancement of cyclin-dependent kinase 2 (CDK2) expression through enrichment at its promoter [142]. LncRNA uc002yug.2 enhanced HIV-1 replication through (1) upregulating expression of Tat; or (2) downregulating messenger RNA (mRNA) of Runt-related transcription factor (RUNX) family member isoforms, which are HIV-1 repressors [143]. An antisense HIV-1-derived lncRNA was shown to recruit chromatin remodeling complexes such as DNMT3A, EZH2, and HDAC1 to the HIV-1 LTR to alter its chromatin state [144]. An antisense HIV-1 transcript formed from the 3' LTR, termed Anti-Sense Protein (ASP), serves the epigenetic function of mediating H3K27me3 deposition by recruitment of PRC2 to the HIV-1 LTR, resulting in decreased RNA Pol II occupancy and HIV-1 transcriptional silencing [145, 146].

## Comparative insights from simpler retroviruses

To contrast these mechanisms in a simpler model, MLV, provides a tractable system in which comparable epigenetic controls can be examined. Having outlined the epigenetic mechanisms controlling HIV-1 latency, we next compare key features of HIV-1 and MLV replication cycles to provide context for their shared and divergent modes of epigenetic regulation.

### Basic comparison of HIV-1 and MLV life cycles

**Fig. 3:**
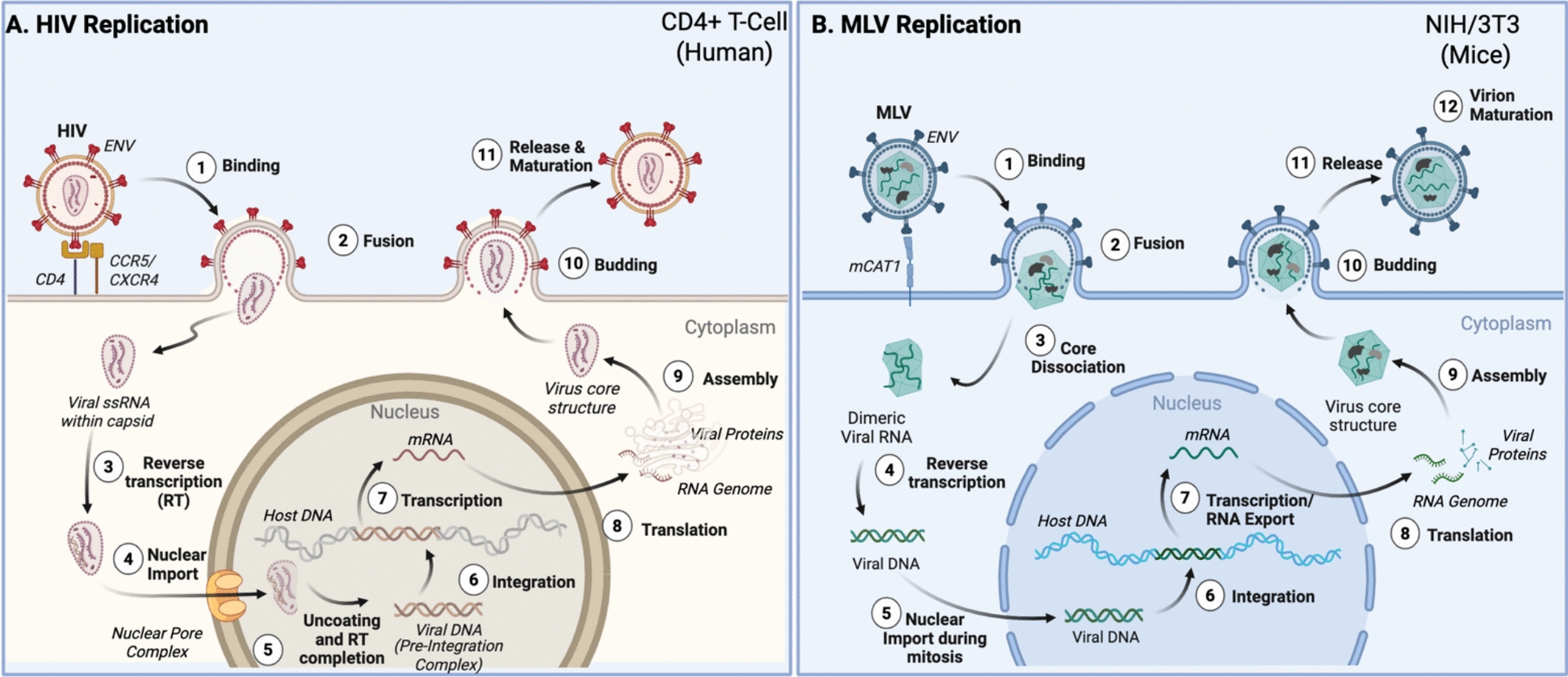
Comparative schematic of HIV-1 and MLV replication cycles and key sites of epigenetic regulation. **A** HIV-1 replication in CD4⁺ T cells [147, 153, 154, 156, 157]. Following receptor engagement and fusion, the viral core is released into the cytoplasm. Reverse transcription (RT) of viral RNA into double-stranded DNA occurs within the intact or partially intact capsid during trafficking toward the nucleus. The capsid interacts with components of the nuclear pore complex, including Nup153 and CPSF6, enabling nuclear import of the core. Reverse transcription and uncoating complete at or within the nucleus, releasing linear viral DNA for integration into host chromatin. Integrated proviruses serve as templates for transcription and translation, producing viral RNAs and proteins that assemble at the plasma membrane, leading to budding and maturation of new virions. **B** MLV replication in NIH/3T3 cells. After entry, the MLV core rapidly dissociates in the cytoplasm, releasing reverse transcription complexes. Reverse transcription initiates in the core but then completes in the cytoplasm, and the resulting linear viral DNA remains associated with viral proteins, including p12, which tethers the pre-integration complex to host chromatin after nuclear entry. For MLV, nuclear entry and integration usually occur during mitosis, when the nuclear membrane disassembles. However, active transport into the nucleus in non-dividing cells has also recently been described. Transcription, translation, and assembly proceed in the cytoplasm, producing progeny virions that bud from the plasma membrane. Epigenetic regulation can occur at multiple stages pre- and post-integration, highlighting both shared and distinct modes of transcriptional control between lentiviruses and gammaretroviruses. *Cellular and viral components are schematic and not represented to biological scale. Image generated with www.biorender.com

MLV is a gammaretrovirus, subfamily oncovirinae type C, single stranded RNA virus often referred to as a “model” of gammaretroviruses. Like HIV-1, which belongs to the lentivirus family, epigenetic mechanisms of viral latency in MLV have been extensively researched. There is a clear difference between these two viruses, resulting from the variation of the location and timing of reverse transcription (Fig. 3). Both viruses include LTRs that serve as promoter regions for viral transcription. However, MLV is called a “simple retrovirus,” as the viral genome only encodes the proteins necessary to assemble the progeny virions (Gag, Pol and Env). On the other hand, HIV-1, referred to as a “complex retrovirus”, encodes two regulatory (Tat and Rev) and four accessory proteins (Nef, Vif, Vpu, and Vpr), in addition to these three genes. MLV was historically believed to require mitosis for replication [7]; however, the necessity for cell division to facilitate nuclear entry by MLV was recently shown to depend on cell type, as active nuclear transport does not occur in fast-dividing NIH 3T3 cells *in vitro*, while evidence suggests that it does in non-dividing primary dendritic cells [147]. HIV-1 can infect and establish latency in nondividing cells as well as dividing cells efficiently, regardless of cell type [7, 9, 148, 149]. The virus can infect both resting and activated CD4 + T cells to establish productive infection and latency [150] and, in fact, exhibits preference for minimally activated cells during latency establishment [151]. In rapidly dividing NIH 3T3 cells, the MLV pre-integration complex (PIC) is unable to enter the intact nucleus [147], whereas the HIV-1 PIC is capable of entering intact nuclei of all cells via active transport through nuclear pores [9, 152, 153]. The location where reverse transcription takes place is also different in these two viruses: MLV is reverse transcribed solely in the cytoplasm, whereas in HIV-1, it begins in the cytoplasm but ends near or in the nucleus (although the precise site of completion remains controversial) [9, 153, 154]. The integration site selection is also different between MLV and HIV-1. MLV shows a strong bias for promoter-proximal integration, leading to efficient reporter expression, whereas HIV-1 integrates into transcriptional units and is limited in expression by its distance from the promoter and the reading frame of the targeted gene [155]. Despite these differences, MLV and HIV-1 are both subject to profound silencing of their unintegrated DNA forms inside the nucleus. The silencing of these viruses is similar in many aspects, but does not require identical host factors.

### Pre-integration latency

Pre-integration HIV-1 latency in T cells was originally believed to be clinically irrelevant because *in vitro* studies suggested unintegrated viral DNA only persisted for no more than one day [158]. However, more recent data demonstrated that unintegrated HIV-1 DNA subspecies persist in CD4 + T cells in PWH treated with integrase inhibitors, and that these subspecies can still integrate upon discontinuation of the drug [159]. Pre-integration latency is believed to be more critical in myeloid cells, as large quantities of unintegrated viral DNA can remain stable and biologically active in non-dividing macrophages for up to two months [160, 161]. It is generally known that unintegrated retroviral DNAs exhibit very low expression, and significant expression levels are attained solely subsequent to the integration of the retroviral DNA into the genome of the host organism [162–166]. The unintegrated DNA forms are rapidly loaded with nucleosomes and heavily silenced by histone modifications [3]. There are specific conserved mechanisms involved in the silencing of the unintegrated retroviral DNAs. Histones bound to the viral DNA were heavily marked by trimethylation of H3 tails on lysine 9 (H3K9me3, a marker of silenced chromatin), and were very low in H3 acetylation in both unintegrated MLV and unintegrated HIV-1 [2, 20]. Whether histone deacetylation or histone methylation is the primary determinant of silencing is not clear. Since the addition of the deacetylation inhibitor, trichostatin A (TSA), can strongly relieve silencing, histone deacetylation might be the major initiating factor leading to silencing. A study by Zhu et al. tried to identify host factors that might mediate the silencing of unintegrated viral DNA by performing an unbiased genome-wide screen using a CRISPR-Cas9 gene knockout (KO) library in the face of integrase-deficient MLV-GFP infection of HeLa cells [1]. They identified the DNA-binding protein Nuclear Protein 220 (NP220), the three proteins (M-Phase Phoshoprotein 8 or MPP8, Transcription Activation Suppressor or TASOR, and Periphilin 1 or PPHLN1) that comprise the HUSH (Human Silencing Hub) complex, and the histone methyltransferase Setdb1 as being required for silencing. They further suggested that NP220 binds directly to viral DNA and acts as a tether to recruit HDACs to remove acetyl groups from nearby histone tails, and to bring the trimeric HUSH complex through interactions with the MPP8 subunit. HUSH has been demonstrated as a restriction factor for HIV-1 [167] and is known to cooperate with Trim28 to repress retrotransposon expression [168]. HUSH is recruited to areas with dense H3K9me3, and loss of the HUSH complex results in decreased H3K9me3 at retroviruses integrated into heterochromatin [169]. The HUSH complex recruits Setdb1 to methylate H3K9, maintaining transcriptional silencing in heterochromatic regions [169], and MPP8 further binds the complex to the DNA by its interaction with the H3K9me3 mark [1]. NP220 is a nuclear double-stranded DNA binding protein known to prefer the sequence CCCCC(G/C). Not all retroviral DNAs are equally silenced by NP220, since existence of its binding site is important for its engagement. The LTRs of both MLV and HIV-1 have matches to these sequences, but there are none in the simple alpharetrovirus Avian Leukosis Virus (ALV), which is not affected by NP220.

Variant histones, such as histone H3.3, are also present in both MLV and HIV-1 unintegrated DNA [2], and are typically involved in condensing nucleosomal DNA into higher-order structures similar to heterochromatin. Both MLV and HIV-1 are silenced by SETDB1 [170–174]. However, there are also evident differences, especially in the response to KO of the silencing machinery. KO of HUSH only relieves the silencing of MLV and has no effect on silencing HIV-1, suggesting other mechanisms are involved in silencing HIV-1 [1, 175, 176]. Additionally, the chaperone proteins Chromatin Assembly Factor 1A/B (CHAF1A/B) were found to mediate silencing of unintegrated HIV-1 DNA, while having no impact on MLV, and this function appeared to be independent of its membership in the CAF-1 complex [177].

Another candidate for silencing unintegrated HIV-1 DNA is the Structural Maintenance of Chromosomes 5/6 (SMC5/6) complex localization factor 2 (SLF2) [175]. Using a genome-wide CRISPR/Cas9 knockout screen, Irwan and colleagues were able to show that SMC5/6 SUMOylates chromatin of unintegrated HIV-1 DNA, and inhibition of SUMOylation prevented epigenetic silencing and promoted transcription of unintegrated DNA [178]. Additional work by Imbert and Langford in microglia also pointed to SMC5/6 as important for latency, but they identified a wide swath of proteins SUMOylated by this complex, showing the post-translational modification was important for many latency-promoting proteins beyond histone modification [179]. Interestingly, YY1 has been suggested as an important silencing factor to the cDNA of the PIC in MLV, but while it was shown to bind to both the MLV and HIV-1 integrase [180], a role in silencing HIV-1 cDNA in PICs has not been reported. However, YY1 binding sites were a consistent finding immediately upstream of HIV-1 integration sites in human lymphocytes and monocyte-derived macrophages from both circulating PBMCs and cord blood [181]. The mechanism of epigenetic silencing of unintegrated HIV-1 episomes requires further exploration, and the exact contribution of pre-integration latency to long-term persistence remains a subject of debate, particularly as the bulk of the work done in this area has been in cell lines and not in primary cells.

### Post-integration latency

The major obstacle in eradicating HIV infection is its latency. While pre-integration silencing reflects early defense against viral DNA, post-integration latency represents long-term epigenetic maintenance of proviral quiescence. The integrated viral genome remains transcriptionally silent for a long period in certain subsets of cells and establishes a persistent infection, despite strong humoral and cellular immune responses against the virus. Understanding the mechanism of silencing and activation of proviral DNA is critical for either of two possible approaches to achieving a functional cure: (1) the complete activation of silent proviruses, as in the “shock and kill” strategy; or (2) their permanent silencing, as in “block and lock” strategy [182]. Unlike HIV infection, MLV infection in permissive cells results in high expression of the integrated viral DNA and rapid production of progeny virus. However, in certain cell types, such as ESCs, hematopoietic stem cells (HSCs), and other developmentally primitive cell types, the proviral DNA at nearly all insertion sites is uniformly silenced [20].

#### KRAB Zinc finger proteins

Latency initiation appears to have a common thread across retroviral classes. Retroviruses have a primer binding site (PBS) that commandeers a host tRNA to initiate reverse transcription. Recent work has highlighted that host mechanisms will target this sequence in the integrated viral DNA for molecular defenses against both endogenous and exogenous retroviral infections, since the PBS sequence must remain conserved to produce functional viral progeny [183, 184]. KRAB zinc finger proteins (ZFPs) are among the main lines of defense, as these factors will bind to PBS sequences of proviruses to recruit silencing machinery (primarily TRIM28/KAP1, SETDB1, and HP1), as described for ZNF304 in HIV-1 above [83]. This mechanism appears species-specific and also viral class-specific, as different classes of retroviruses will rely on complementarity of PBS sequences to various cellular tRNAs [183, 185]. The silencing of MLV in mouse ES cells is initiated by the ZFP ZFP809 and YY1, which recruit a large protein complex with a central component of Trim28/Kap1 to induce silencing. Similar to HIV-1, murine Trim28/Kap1 acts as a scaffold to recruit HDACs, the histone methyltransferase Setdb1, the methylated histone binding protein HP1, and ultimately DNMTs responsible for *de novo* DNA methylation [171–174]. Further work in cell lines has suggested that HP1 is an absolute requirement for MLV restriction [186], but while the essential role of Setdb1 in enMLV silencing was reiterated by Maksakova and colleagues [187], HP1 as an absolute requirement for silencing was disputed. TRIM28/KAP1 can also autoSUMOylate, which is required for additional recruitment of the NuRD complex [188]. ZFP961, another mouse ZFP targeting PBS-Lys, is able to silence the endogenous retrovirus (ERV)-K subgroup of ERVs in mouse ESCs [183].

Further ZFPs targeting endogenous retroviruses include ZNF417 and ZNF587, which bind to the PBS-Lys of the Human Endogenous Retrovirus-K (HERV-K, a betaretrovirus) for repression [189], and recent work shows these two ZFPs inhibit HIV-1 as well [183]. ZBRK1 was also shown to repress the HIV-1 LTR [190], and ZNF10 interacts with NFkB and Specificity protein 1 (Sp1) motifs in the HIV-1 LTR to recruit TRIM28/KAP1 [190]. Additionally, ZNF506 appears important for targeting the PBS-Pro sequence for HERV suppression, as well as human T-cell lymphotropic virus (HTLV) [184, 191].

HIV-1 is well-documented to exhibit clonal expansion related to the expansion of the memory CD4 + T cell pool, discussed in [192]. Interestingly, genomically intact clonally expanded HIV-1 proviruses are overrepresented in KRAB zinc finger genes, likely because these genes are downregulated upon CD4 + T cell activation, promoting latency of the provirus while expanding the T cell pool [193]. Intact proviral integrations of HIV-1 in KRAB ZFPs were shown to be especially common in elite controllers, suggesting these integration sites could contribute to HIV-1 control [194]. Recently, suppression of KRAB ZFPs regulating human transposable elements flanking innate immune genes was shown to be important in HIV-1 regulation by elite controllers, suggesting a potential reason for this finding and illustrating the complexity of the interplay between KRAB ZFPs with endogenous and exogenous retroviral elements [195].

In addition to proviral suppression, some ZFPs can also target viral RNA for degradation as described for the Zinc Finger Antiviral Protein (ZAP) protein. ZAP targets not only retroviruses (including both MLV and HIV-1, as well as ALV and HTLV), but a host of other virus types, including flaviviruses, filoviruses, togaviruses, coronaviruses, etc. [196, 197].

#### Retroviral silencing machinery

Following ZFP-mediated recruitment of silencing machinery, including DNMTs, proviral MLV DNA becomes methylated on cytosine residues, resulting in its transcriptional silencing [198]. Setdb1 was initially determined to be critical for H3K9me3 deposition and silencing of ERVs in mouse ESCs, but not in differentiated fibroblasts [199]. Elimination of Setdb1 in murine B lymphocytes resulted in loss of H3K9me3 and DNA methylation at ERV LTRs concomitant with derepression of the proviruses, but this was not a consistent finding for all ERVs [200]. The same was true in mouse ESCs, although loss of DNA methylation was more prominent [201]. Collins et al*.* concluded that the impact of this derepression was variable depending on cell type, as transcription factors important for LTR activation were differentially expressed [200]. Interestingly, G9a was previously shown to be critical for silencing newly integrated MLV, but downstream maintenance of H3K9 methylation required the H3K9 methyltransferase Setdb1 exclusively in murine ESCs. Additionally, G9a was also required for establishment of DNA methylation of the newly integrated MLV provirus [202].

The data on the overall impact of histone modifications in gammaretroviruses are limited, especially in primary somatic cells. As was recently reported for HIV-1, RYBP and H2AK119ub were shown to play a role in class III murine ERV silencing in ESCs, although not for class I ERVs known to contain gammaretroviruses [203]. Rather, this mechanism appears to be an alternative to Setdb1 and H3K9 methylation-based silencing mechanisms typical for endogenous MLVs and other class I ERVs [204]. However, Mikkelsen and colleagues reported that endogenous MLVs contained silencing-associated H3K9me3 and H4K20me3 in ESCs, but not in murine fibroblasts [205], and Matsui et al*.* showed further that Setdb1 was required for both H3K9me3 and H4K20me3 in the enMLV provirus of ESCs, suggesting that as for HIV-1 [69], Setdb1 acted upstream of Suv4-20h1 and Suv4-20h2 homologs responsible for H4K20 trimethylation [199]. Wolf and colleagues found that both H3K9me3 and H4K20me3 were also located within Porcine Endogenous Retrovirus (PERV) proviruses in both ESCs and in PK15 cells derived from pig kidney [206]. LaMere et al*.* also reported that endogenous MLV in ESCs and neuroprogenitor cells can be marked by H3K4me3, H3K9me3, H3K27me3, H3K27ac, H3K9ac, and H3K36me3 [207], but histone modification data for exogenous gammaretroviruses are especially scarce.

Stephen Goff's group has published most extensively on gammaretroviral (particularly MLV) histone modifications. One noteworthy study from Dr. Goff's lab found that two different silencing systems appear to be in place leading to extensive H3K27me3 and H3K9me3 marks and decreased H3 acetylation in LTRs of MLVs in murine ESCs and F9 embryonic carcinoma cells: a highly efficient PBS-dependent and a less efficient PBS-independent mechanism. They also concluded that machinery leading to repression of endogenous and exogenous MLVs was highly synonymous [208]. Of note, the silencing mechanisms found in ESCs and F9 cells appeared completely non-operational in the differentiated NIH 3T3 cell line. Importantly, several studies showed that DNA methyltransferase 3-like (DNMT3L), which has no catalytic activity of its own [209], is a critical mediator of MLV silencing in ESCs, interacting with Trim28/Kap1 to recruit silencing machinery, including HDAC1, SETDB1 and DNMT3A [198, 210]. Recruitment of these modulators resulted in increased H3K9me3 and *de novo* DNA methylation at the MLV LTR. DNMT3L is not ordinarily expressed in somatic cells, yet ectopic expression of DNMT3L in somatic cells resulted in powerful recruitment of these repressive enzymes [210], and binding of DNMT3L to chromatin was mediated by unmethylated H3K4 [198]. More recent work stemming from this finding showed that ectopic expression of DNMT3L in late-passage mouse embryonic fibroblasts (MEFs) helped delay senescence via induction of H3K9me3-mediated silencing of derepressed ERVs and transposons [211]. To date, this mechanism appears completely unexplored in the context of HIV, likely because of the virus' restriction to somatic cells. Another recent finding in endogenous and exogenous MLV silencing in murine ESCs was the deposition of histone variant H3.3 onto integrated proviruses, which was a requirement for transcriptional silencing and shown to be mediated by Trim28 upstream of Setdb1 [212, 213]. H3.3 deposition on repetitive elements was found to be facilitated by the Death Domain Associated Protein (Daxx/Atrx) complex in mouse ESCs [214]. As discussed for pre-integration complexes, this histone variant is also present in unintegrated MLV and HIV-1 DNA [2], suggesting its presence is a defense against retroviral genome invaders and potentially should be explored as a mechanism for silencing integrated HIV-1 DNA as well.

Screening genome wide small interfering RNA (siRNA) libraries has revealed additional factors that are required for MLV silencing, such as histone chaperones (Chaf1a/b) and SUMOylation factors (Sumo2/Ube2i/Sae1/Uba2/Senp6) [42]. Unsurprisingly, the CAF-1 complex containing CHAF1A and CHAF1B subunits and SUMOylation machinery were recently found to play a role in HIV-1 latency, as well [215], and at the time of this writing, CHAF1A was shown to be a prospective therapeutic target for reactivating HIV-1 in the context of a "shock and kill" approach [216]. Other clues have been found in studying endogenous retroviruses, such as Avian Sarcoma Virus (ASV) and FeLV. In human cell lines infected with ASV, the histone chaperone Daxx plays a key role by interacting directly with the ASV integrase (IN), resulting in silencing of integrated viral DNA via recruitment of HDACs [217–219], which has also been shown true for HIV-1 [220]. Murine Daxx was important for histone variant H3.3 deposition on murine endogenous elements [214]. Interestingly, human DAXX restricted HIV-1 infection via reverse transcriptase inhibition in the cytoplasm, but its defined HIV-silencing mechanisms did not relate back to its histone chaperone activities as for the simpler retroviruses [221, 222]. Instead, it appeared to prevent HIV-1 uncoating and its associated reverse transcription in a SUMO-interacting motif (SIM)-dependent manner [222].

Surprisingly, BET proteins play a somewhat different role for gammaretroviruses such as MLV. Instead of serving to activate or repress proviral expression, BRD2, BRD3, and BRD4 interact with the viral integrase to tether the pre-integration complex to acetylated histones, directing the provirus toward integration into GC-rich promoters and enhancers [223–226]. However, as yet, no reports have specified the impact of BET proteins upon transcriptional activity of MLV once integrated.

Non-coding RNAs regulating gammaretroviruses are poorly characterized compared to HIV-1. Overall, gammaretroviral RNA spends less time in the cytoplasm dissociated from Gag proteins, leading to speculation that it might be less prone to miRNA regulation. MLV maintains two cytoplasmic unspliced viral RNA pools: one used for viral protein translation and the other packaged as genomic RNA into virions. By contrast, HIV-1 maintains a single pool of unspliced genomic RNA that serves both as the mRNA for Gag/Pol translation and as the packaging template, distinct from the spliced RNA species used exclusively for protein production [13]. Descriptions of host-derived non-coding RNAs regulating gammaretroviruses are non-existent in the literature, but there are a few cases of virus-derived non-coding RNAs. In FeLV, miRNA transcription is linked to endogenous solo LTRs distributed within the domestic cat genome, with potential impacts on domestic cat exogenous FeLV susceptibility and pathogenesis [227]. Additionally, while reports of lncRNAs for gammaretroviruses are limited, one study has found that a lncRNA was derived from the murine ERV1 family, which is closely related to MLV. This lncRNA was found to promote intracellular antiviral responses by depressing the RELA subunit of NFkB [228]. A similar mechanism was found for the deltaretrovirus Bovine Leukemia Virus (BLV), where the BLV-derived lncRNA AS1-S was found to alter RNA binding protein interactions with cellular mRNAs, potentially leading to cellular proliferation and neoplastic processes characteristic of bovine leukosis [229]. Antisense transcription of other types of exogenous retroviruses has also been recognized, including MLV [230], as well as lentiviruses FIV [231] and Bovine Immunodeficiency Virus (BIV) [232], but the retroviral transcripts that have been characterized in other species appear to perform modulation of host transcripts rather than act on proviral expression itself. Finally, HTLV generates an antisense transcript of the gene HBZ, which has repressive functions independent of HBZ protein translation and is believed to be a key player in HTLV silencing by blocking transcriptional machinery at the 5' LTR [233–235]. However, this viral RNA also increases proliferation of HTLV-infected cells, ensuring its propagation despite its transcriptional silencing [233].

### KoRV endogenization and its implications for understanding mastery of retroviral infections

Studying recently endogenized retroviruses provides unique insight into host mechanisms of post-integration silencing that could inform strategies for controlling HIV-1 latency. While HIV and other lentiviruses rarely endogenize [236], approximately 8% of the human genome is comprised of ancient endogenized retroviral infections. Multiple bodies of work have hypothesized that the epigenetic drivers that silence HERVs/ERVs can highlight host repression pathways that might also modulate HIV-1 proviral transcription and latency, thereby identifying candidate mechanisms that could be leveraged to limit HIV-1 transcription [167, 237–239]. Indeed, several mechanisms known to silence HERVs have already been shown to play a role in HIV-1 silencing [168, 200]. An especially informative case study is the koala retrovirus KoRV, a gammaretrovirus currently in the process of endogenizing, which bridges the gap between exogenous and endogenous retroviruses and highlights novel mechanisms of transcriptional repression mediated by piRNAs, which could also inform control of the latent HIV-1 provirus.

KoRV is believed to have descended from an Asian rodent retrovirus [240]. A few variants have been described based on different envelope sequences, but the primary subtype that has endogenized is KoRV-A. Yu and colleagues reported in 2019 that KoRV-A promotes an innate molecular response to a new genome invasion that is driven primarily by unspliced viral transcripts [241]. A resulting sense-strand piRNA response causes cis silencing of proviral transcription. However, over time, adaptive molecular immunity through cluster insertions has resulted from continued transpositions, silencing the endogenous element through trans-acting anti-sense piRNAs. For KoRV-A, virus-specific piRNAs were identified only in the testis, suggesting this innate mechanism is restricted to germ cells and not a method of retroviral control in somatic cells. Aside from guiding Argonaute proteins to the transposable element for post-transcriptional RNA degradation, piRNA-loaded PIWI proteins are imported into the nucleus to initiate histone and DNA methylation, resulting in co-transcriptional repression [242, 243]. Further work by Yu and colleagues suggested these mechanisms are at play for koalas north of the Brisbane River where KoRV-A first began to endogenize, as the KoRV-A LTR was significantly more methylated in germ cell samples from animals in this region compared to those from the south, where KoRV-A is not fully endogenized [243]. Further, they found that koalas in the north had an anti-sense KoRV-A insertion that produced repressive anti-sense piRNAs, guiding transcriptional silencing and re-shaping the koala genome [243]. Recent literature also points to a similar response to ALV in domestic chickens. ALV endogenization is less recent than that of KoRV, but an exogenous virus still circulates, and one particular endogenous integrant was shown to be protective via piRNA production [244]. These findings demonstrate how post-integration transcriptional silencing can be rapidly established upon genome invasion, providing a conceptual parallel to latent HIV-1 proviruses.

Historically, piRNAs were thought to be expressed only in germ cells; however, recent evidence has come to light suggesting they are also expressed in highly differentiated somatic cells [114, 132], highlighting the potential for piRNA-mediated regulation of exogenous retroviruses, including HIV-1, which remains largely unexplored. As described above, two different PIWI proteins have been found to regulate HIV-1 [133, 134], but both seem to be piRNA-independent, and PIWIL2 in particular was shown to interact with tRNAs instead [134]. One might speculate that perhaps piRNAs are not widely expressed in CD4 + T cells, but piRNAs have been characterized and shown to be differentially expressed in lymphocytes in the context of other viral infections, such as SARS-CoV-2 [245], which suggests further research into piRNAs in exogenous retroviral infections is warranted. Collectively, the findings from MLV and KoRV provide a comparative perspective on epigenetic silencing in simpler retroviruses. Together, these mechanisms illustrate both conserved and virus-specific layers of epigenetic control.

## Learning from animal models of lentiviral infection: FIV and SIV

### Proviral epigenetic regulation of non-human lentiviruses

Little has been published on epigenetic mechanisms of lentiviral control in non-human species, and most studies in the literature are focused on epigenetic pathways impacting host genes rather than those directed at integrated or unintegrated viral DNA. The two best-studied lentiviral infections in animals are FIV and SIV, as these two viruses best model the pathogenesis of HIV in humans [246].

Like SIV and HIV, FIV originated in Africa and is endemic in a number of free-ranging feline species. It was first discovered by Neils Pedersen in domestic cats in 1984 and is thought to have entered the domestic feline population relatively recently, although no specific time line for the virus' emergence has been proposed during the era since cats were domesticated approximately 10,000 years ago [247]. Also like HIV and SIV, FIV infects CD4 + T cells and macrophages. However, its tropism is broader, allowing it to infect CD8 + T cells and B cells as well [248], as its primary receptor is CD134 rather than CD4 [249]. McDonnel et al*.* explored histone modifications associated with the proviral promoter during latency and following *in vitro* reactivation using ChIP-qPCR of the 5' LTR and discovered a decrease in histone acetylation (H3K9ac and H3K14ac) and increased repressive H3K27me3 when compared to reactivated proviruses [250]. The same year, Murphy and colleagues examined LTR DNA methylation of the FIV provirus using bisulfite PCR in a latency model of cells from experimentally infected animals and found no evidence of promoter hypermethylation during latency in either CD4 + T cells or monocytes [251].

Infection of Asian macaques with SIV results in fulminant immunodeficiency reminiscent of the course of HIV in humans. Similar to the FIV LTR, the SIV LTR has also been shown to be de-acetylated and exhibit increased histone methylation during latency [252, 253]. One study by Barber et al*.* showed macaque models with SIV had an increase in histone H4 acetylation at the LTR during the acute infection stage, which contributed to production of full-length viral RNA in the brain with the help of liver-enriched transcriptional activator protein (LAP) [252]. All the while, an increase in liver-enriched transcriptional inhibitory protein (LIP) accompanied by de-acetylation of H4 was characteristic of SIV latency in the central nervous system (CNS), which implied that chromatin remodeling through histone acetylation can actively contribute to the pathogenesis of SIV in the brain [252].

### Epigenetic regulation of T cell control

Beyond proviral chromatin regulation, host immune states such as T-cell activation further influence epigenetic silencing and reactivation. SIV infects African green monkeys (AGM) and sootey mangabey monkeys (SM) with few or no clinical signs, likely because this virus has been endemic to African primates for an estimated 32,000 years and is believed to have co-evolved with African primate species. Although limited studies show epigenetic control of the provirus [252, 253], SIV remains replication-competent in African species, so mechanisms of control appear to be directed more toward host tolerance rather than transcriptional control of the provirus itself [254]. Therefore, examination of host mechanisms for viral silencing in African primates provides insights into strategies for HIV vaccination and eradication. Recently, Mudd and colleagues demonstrated that AGMs systemically downregulated CD4 upon infection with SIV, decreasing the pool of available cells for infection with the virus, and this response was concomitant with atypical regulation of DNA methylation machinery and methylation of the CD4 promoter, suggesting a potential epigenetic mechanism directed toward host genes in the face of infection [255]. Jochems and colleagues further examined genome-wide DNA methylation in CD4 + T cells during the course of both pathogenic SIV infection in macaques and non-pathogenic infection in AGMs and reported that SIV infection in macaques was associated with differential methylation in genes related to Th1 signaling, while SIV infection in AGMs was associated with differential methylation in genes coding for regulatory proteins, both in blood and in lymph nodes [256].

CD8 + T cells' ability to travel to sites of viral replication in the lymph nodes (LN) is pivotal to the immune response in chronic SIV/HIV infection [257]. In an effort to characterize CD8 + T cell dysfunction during lentiviral infection, epigenetic studies have been performed on virus-specific CD8 + T cells during both SIV and FIV infection [258]. Binding of Forkhead box P3 (Foxp3) to Interleukin 2 (IL-2), Interferon gamma (IFN-γ), and Tumor Necrosis Factor alpha (TNF-α) promoters in CD8 + T cells in response to lentiviral activation of regulatory T cells (Tregs) during FIV infection was epigenetically modulated via DNA demethylation and histone acetylation [246, 259, 260]. In cats with FIV, Foxp3 + Treg cells exhibited miRNA10a upregulation, which appeared to stabilize Foxp3 expression in these cells *ex vivo* [261]. Surprisingly, less work has been done to characterize the host epigenetic response to SIV infection [246]. Ferrando-Martinez et al*.* proposed a substantial increase in total follicular CD8 + T cells in lymph nodes during early chronic and pathogenic SIV infection in nonhuman primates along with tissue inflammatory mediators [257]. One report identified a potential role for EZH2-mediated methylation of H3K27 in B and T follicular helper cells (Tfh) in the germinal centers during SIV infection [262]. They suggested that abnormal GC follicular helper T cell B cell differentiation and maturation can play a role in the establishment of viral reservoirs during SIV infection and cause latency [262]. Further work by Rahmberg et al*.* used longitudinal samples of peripheral naive and memory CD4 + and CD8 + T cells from two macaque species before, during, and after SIV infection to show that SIV infection led to significant changes to chromatin accessibility and the transcriptome, which were only partially reversed by ART [263]. However, our overall understanding of the role of epigenetics during lentiviral infections in animals is under-studied compared to that of HIV in humans. This dearth of information in the literature is surprising in light of the fact that Asian macaques with SIV and domestic cats with FIV serve as animal models for HIV, and the proposed cure strategies for HIV center around epigenetic modulation of integrated HIV-1 DNA [264].

## Conclusions and future directions

While HIV-1 is better-studied than any single retrovirus in the animal kingdom, the comparisons and contrasts to what we know about other types of retroviruses can provide some guidance on possible therapeutic strategies to explore, or minimally broaden our understanding of HIV latency. Table 3 provides comparisons of prominent silencing mechanisms described here between HIV-1 and the gammaretroviruses. Hints have already been taken from the natural host response of AGMs to SIV, which uses the widespread downregulation of CD4 to undercut the virus' ability to infect cells. While CD4 in human systems is likely too critical to the adaptive immune response to manipulate, the very first successful functional cures of HIV-1 came from bone marrow transplants from donors who carried a C–C motif Chemokine Receptor 5 (CCR5) mutation, which is an important HIV-1 co-receptor. However, bone marrow transplants are inherently risky and typically only reserved for treatment of neoplastic processes, and the CCR5Δ32/Δ32 mutation is present in only 1% of the population, making it difficult to locate HLA-matched donors with the homozygous mutation [265, 266]. More recent approaches seek to use CRISPR/Cas9 manipulation of CCR5 on HSCs, widening the pool of available prospective donors [267].

**Table 3:** Basic epigenetic mechanisms acting directly on viral DNA or viral RNA pre- and post-integration

		Gammaretrovirus			Lentivirus		
	Factor/modification	Cell line	Primary cells	References (PMID)	Cell line	Primary cells	References (PMID)
Pre-integration	YY1	Role unclear	No data	20519390	No data	Role unclear	19100594
	H3K9me3	Repressive	No data	30487602	Repressive	No data	31685613, 22082156
	HUSH complex	Repressive	No data	30487602	No impact	No data	36376394
	CHAF1A/B	No effect	No data	35074917	Repressive	No data	35074917
	NP220	Repressive	No data	30487602	No impact	No data	30487602
	SETDB1	Repressive	No data	30487602	Repressive	No data	22082156
	SMC5/6	No data	No data	N/A	Repressive	Repressive	33811831, 36376394, 39937027
	Histone H3.3	Repressive	No data	31685613	Repressive	No data	31685613
Post-integration	KRAB Zinc finger proteins	No data	Repressive	19270682, 17923087, 35259018, 37697430, 24162661, 31518518, 32923624, 35979358, 23154467, 26365490	Repressive	Repressive	32956422, 22975076, 26096782, 34636876, 32848246
	YY1	No role in differentiated cells	Repressive	23810560, 29407374, 1309593	Active or Repressive	No data	31818358, 10888618, 24116200, 8289393, 9371597, 11940,654
	TRIM28/KAP1	No data	Repressive	17923087, 20075919, 27795446, 23154467, 24991018, 26365490, 27866901, 23810560, 29407374	Active or Repressive	Active	26725010, 30943398, 30652970, 30670656, 38257816, 26096782
	SETDB1	Repressive	Repressive	30487602, 20164836, 21624812, 26100872, 30737147, 29703894, 21774827, 39476839, 27795446, 24991018, 26365490, 27866901	Repressive	No data	32956422, 32161174, 26096,782, 22082156
	HP1	Repressive	Repressive	20164836, 18287239, 23735015, 21774827, 24991018	Repressive	Repressive	17245431, 32956422, 28494238, 17245432, 28494238, 32161174, 26096782
	SUV39H members	Repressive	Repressive	23735015, 21427230	Repressive	Repressive	20335163, 28246360, 23541084, 17245432, 22020221, 17245431
	CAF-1, Chaf1a/b	No data	Repressive	26365490, 27795446	Repressive	Repressive	33739466, 41334912
	BET proteins	Facilitates integration, but no data on chromatin modulation	No data	35852337, 24183673, 23818621, 24049186, 24623816	Usually Repressive, but active in some scenarios	Repressive	40402245, 33413475, 28844864, 29684085, 32188727, 31329163, 23517573
	HAT/Histone acetylation	Active	Active	23154467, 24991018, 27866901	Usually active, but repressive with BRD4	Active	9733796, 9811832, 14657027, 31999790, 38134881, 24620025, 10888618, 29684085
	HDAC	Repressive	Repressive	24991018, 26365490, 10629041, 16603064	Repressive	Repressive	19557157, 10888618, 38134881, 17245431, 19239360, 24620025, 23517573, 11940654, 17670825, 19279091, 20846395, 25136952, 26041287, 22067449, 31888084, 38134881
	H3K9me3	Repressive	Repressive	20164836, 30487602, 26100872, 20164836, 21624812, 29703894, 21774827, 23324470, 39476839, 27795446, 23154467, 24991018, 26365490, 27866901	Repressive	Repressive	32161174, 28246360, 31999790, 35797416, 35319230, 17245432, 22020221, 17245431, 20335163
	PRC2/H3K27me3	Repressive	Repressive	27795446, 23154467, 20123906	Repressive	Repressive	28246360, 31999790, 35797416, 35319230, 21715480, 26041287, 25572573
	H2AK119ub		Repressive	37604781, 22269950	Repressive		32956422, 40361117, 21496352, 24642637
	DNMT/cytosine methylation	Repressive	Repressive	12370304, 12036582, 6183444, 20164836, 26100872, 21624812, 21427230, 1702844, 14559924, 23154467, 24991018, 10629041	Repressive	Role unclear	9557157, 19696893, 2323336, 22345448, 26900410, 22973038, 24418551, 31519219. 19557157, 11559808, 30670613
	H3K4me3	No data	Active	26365490	Active or Repressive	Active	31999790, 40361117, 20227666, 30105631, 35319230, 15564463, 22067449
	H3K36me3	No data	No data	N/A	Active	Limited data	31999790, 19141475
	H4K20me1	No data	No data	N/A	Repressive	No data	28494238
	H4K20me3	No data	Repressive	20164836, 17603471, 21774827, 23324470	Repressive	No data	33739466
	Histone H3.3	No data	Repressive	25938714, 37170146	No data	No data	N/A
	DAXX	Repressive (ASV)	Repressive (ERVs)	18094192, 15795247, 23221555, 26340527	Repressive	No data	18558084, 26566030, 32545337
	miRNA	No data	Silencing	34495702	Silencing	Silencing	25486977, 19560422, 17906637
	lncRNA	No data	ERV-derived host gene silencing	31363026	Silencing or activating	Silencing or activating	32408053, 32581100, 31710657, 30788509, 31551335, 29491162, 23362321, 30036787, 25728138, 27291871, 33086748
	piRNA	No data	Repressive	21546553, 40056902	Silencing (PIWI proteins only)	Silencing (PIWI proteins only)	32161174, 28331090

On its surface, variant histone H3.3 is an attractive target to examine. As described above, work in MLV has shown its importance in both pre- and post-integration transcriptional control [2, 212, 213], and it has already been shown as important for silencing HIV-1 in PICs as well [2], calling into question whether this might also be a prospective target for post-integration silencing of HIV-1. However, as is true for many epigenetic-based therapeutics, manipulation of H3.3 would likely need to be targeted specifically to infected cells, as instability in either of the two genes encoding it can result in substantial human disease, including high-grade neoplasia [268, 269].

While histone H3.3 might be a poor target for therapeutics due to potential for off-target effects, the role of DAXX in HIV-1 infection is possibly worth further exploration. This histone chaperone appears to be important for murine repetitive element silencing via H3.3 recruitment [214]. Examination of its silencing role in HIV-1 infection has been focused more upon viral uncoating and reverse transcription [220–222], but potential for DAXX inhibition as an HIV-1 latency reversing agent has never been investigated. However, DAXX inhibitors have been developed for use as possible therapeutics against multiple types of cancers [270, 271], making them attractive candidates to explore as HIV-1 latency reversing agents.

DNMT3L is another strong silencing molecule in germ cells that targets MLV; however, its expression in somatic cells is rare, and a search of RNA-seq data yields no evidence that it is upregulated in the face of HIV infection. Its expression has also been associated with certain cancers [272], but potentially protective in others [273]. Ectopic expression of DNMT3L *in vivo* is likely to be challenging, but DNMT3L impacts upon lentiviral expression is one area that has not been previously examined in HIV and potentially deserves further exploration.

Finally, piRNAs are not reported as mechanisms of latency against HIV-1 at all, but they are for other simple retroviruses, such as KoRV [241, 243] and ALV [244]. Non-coding RNAs are an area of active study in HIV expression and latency [274], but the focus has understandably been more upon miRNAs and lncRNAs that are well-documented to impact HIV-1 expression and are expressed in cell types targeted by HIV-1. PiRNAs probably have less potential as therapeutics directed toward HIV-1, but it is an open question whether they might play a role in HIV-1 silencing, and if they do, whether there is any way to target the machinery surrounding piRNA-mediated HIV-1 silencing.

Summarily, natural infections with more rudimentary retroviruses, whether endogenous or exogenous, might be able to inform on other areas to examine in the search for an HIV cure, whether through "block and lock" or "shock and kill" mechanisms. Many natural cellular defenses toward retroviruses are specific to germ cells or ESCs, making those processes less attractive as therapeutic targets in HIV infection. However, we argue that they provide insight into mechanisms of retroviral latency that are still applicable toward HIV and might illuminate other strategies to explore. Additionally, there is a surprising dearth of information about pre-integration latency, despite the fact that PICs are notoriously silenced in HIV-infected cells, calling into question whether more thorough examination of the mechanisms surrounding pre-integration latency could provide other targets to consider post-integration.

## Data Availability

No datasets were generated or analysed during the current study.

## Abbreviations

HIV: Human immunodeficiency virus
ART: Antiretroviral therapy
MLV: Murine leukemia virus
HERV: Human endogenous retrovirus
DNA: Deoxyribonucleic acid
RNA: Ribonucleic acid
ESC: Embryonic stem cell
KRAB: Krüppel-associated box-containing
SIV: Simian Immunodeficiency Virus
FIV: Feline Immunodeficiency Virus
DNMT: DNA methyltransferase
5mC: 5-Methylcytosine
CGI: CpG Island
LTR: Long terminal repeat
MBD: Methyl-CpG binding domain
HDAC: Histone deacetylase
NuRD: Nucleosome remodeling and recruiting the histone deacetylation
PWH: People with HIV
hGFP: Humanized green fluorescent protein
PCR: Polymerase chain reaction
SWI/SNF: SWitch/Sucrose Non-Fermentable
BAF: BRG1/BRM-Associated Factor
PBAF: Polybromo-associated BAF
SUMO: Small ubiquitin-like modifier
HAT: Histone acetyltransferase
CBP: CREB-binding protein
GCN5: General control of amino acid synthesis protein 5-like
P/CAF: P300/CBP Associated Factor
KAT2B: Lysine acetyltransferase 2B
YY1: Ying-yang factor 1
CTIP2: COUP-TF interacting protein
BET: Bromodomain and extraterminal motif
BRD: Bromodomain containing
P-TEFb: Positive transcription elongation factor b
KAT5: Lysine acetyltransferase 5
CDK9: Cyclin-dependent kinase 9
me: Methylation
EZH2: Enhancer of Zeste Homolog 2
PRC: Polycomb repressive complex
EHMT: Euchromatic histone lysine methyltransferase
UTX-1: Ubiquitously transcribed X-chromosome 1
SUV39H: Suppressor of Variegation 39 Homolog
CEBPb: CCAAT Enhancer Binding Protein beta
KLF: Krüppel-like factor
P53: Tumor protein 53
SETDB: SET domain bifurcated
HP1a: Heterochromatin protein 1-alpha
PBMCs: Peripheral blood mononuclear cells
CRISPR: Clustered regularly interspaced short palindromic repeats
Cas9: CRISPR-associated protein 9
ZNF: Zinc finger nuclease
TRIM28: Tripartite motif containing 28
Nurr1: Nerve growth factor 1B-like receptor
RYBP: RING1 and YY1 binding protein
LEDGF: Lens epithelium derived growth factor
SETD2: SET domain containing 2
MINA53: Myc induced nuclear antigen 53
RNAi: RNA interference
SET: Su(var)3–9, enhancer-of-zeste, and trithorax
MYND: Myeloid, nervy, and DEAF-1 domain-containing
SMYD: SET and MYND domain containing protein
L3MBTL1: Lethal malignant brain tumor-like protein 1
LINE: Long interspersed nuclear element
TAR: Trans-activation response
LSD1: Lysine-specific histone demethylase 1
CARM1: Co-activator of arginine methyltransferase 1
PRMT: Protein arginine methyltransferase
WDR5: WD repeat domain 5
MLL: Mixed lineage leukemia
ACSS2: Acyl-CoA synthetase short chain family member 2
miRNA: MicroRNA
piRNA: Piwi RNA
lncRNA: Long non-coding RNA
RISC: RNA-induced silencing complex
SAHA: Suberoylanilide hydroxamic acid
FOXO1: Forkhead box O1
PAZ: PIWI-AGO-Zwille
*NKILA*: NFkB-interacting lncRNA
*PVT1*: Plasmacytoma variant translocation 1
*MALAT1*: Metastasis-associated lung adenocarcinoma transcript 1
*HEAL*: HIV-enhanced lncRNA
*NRON*: Nuclear factor of activated T cells
*NRIR*: Negative regulator of interferon response
LRA: Latency reversing agent
FUS: Fused in sarcoma
CDK2: Cyclin-dependent kinase 2
mRNA: Messenger RNA
RUNX: Runt-related transcription factor
NFAT: Nuclear factor or activated T cells
CUL4B: Cullin 4B
ASP: Anti-sense protein
PIC: Pre-integration complex
TSA: Trichostatin A
KO: Knockout
NP220: Nuclear protein 220
MPP8: M-Phase phoshoprotein 8
TASOR: Transcription activation suppressor
PPHLN1: Periphilin 1
HUSH: Human silencing hub
CHAF/CAF: Chromatin assembly factor
SMC: Structural maintenance of chromosomes
SLF2: SMC5/6 complex localization factor 2
PBS: Primer binding site
tRNA: Transfer RNA
ZFP: Zinc finger protein
ERV: Endogenous retrovirus
Sp1: Specificity protein 1
HTLV: Human T-cell lymphotropic virus
ZAP: Zinc finger antiviral protein
ALV: Avian Leukosis Virus
siRNA: Small interfering RNA
ASV: Avian sarcoma virus
FeLV: Feline leukemia virus
IN: Integrase
BLV: Bovine leukemia virus
BIV: Bovine immunodeficiency virus
PERV: Porcine endogenous retrovirus
DNMT3L: DNA methyltransferase 3-like
MEF: Mouse embryonic fibroblast
KoRV: Koala Retrovirus
AGM: African green monkey
SM: Sootey mangabey
LAP: Liver-enriched transcriptional activator protein
LIP: Liver-enriched transcriptional inhibitory protein
CNS: Central nervous system
Foxp3: Forkhead box P3
IL-2: Interleukin 2
IFN-γ: Interferon gamma
TNF-α: Tumor necrosis factor alpha
Treg: Regulatory T cell
Tfh: T follicular helper
CCR5: C–C motif chemokine receptor 5
HSC: Hematopoietic stem cell
